# Measuring attitude towards mathematics using Likert scale surveys: The weighted average

**DOI:** 10.1371/journal.pone.0239626

**Published:** 2020-10-01

**Authors:** Carmen León-Mantero, José Carlos Casas-Rosal, Cristina Pedrosa-Jesús, Alexander Maz-Machado

**Affiliations:** 1 Department of Mathematics, University of Córdoba, Córdoba, Spain; 2 Department of Statistics, Econometrics, Operational Research, Business Organization and Applied Economics, University of Córdoba, Córdoba, Spain; Aalborg University, DENMARK

## Abstract

In the research on mathematics education, numerous Likert-type instruments estimating attitudes toward mathematics are sometimes composed of factors with a high correlation, which can make it difficult to assign the statements from the scale to each estimated factor. Further, the measurement of attitudes is usually done by adding the scores but ignoring the existence of possible differences in the importance that each item can have in its factor estimation. A revision of the methodology for the study of attitudes toward mathematics for the correct validation of the instrument is carried out in this research, and an estimation of the factors of attitude is proposed using the calculation of the weighted average of scores based on the importance that each item has in the explanation of its factor, which is given by a structural equation model. This methodology has been applied to Auzmendi’s scale of attitudes toward mathematics measurement in a sample of 1293 university students. The factors were estimated using simple and weighted averages; significant differences have been obtained in the measurements, as well on those shown with the organization proposed by Auzmendi.

## Introduction

Among researchers who focus on the influence of the affective domain in the teaching and learning of mathematics, it is assumed that cognitive factors are not the only determining ones. When students and teachers work on mathematics or other subjects, their interests, beliefs, feelings, and attitudes influence play an important role in this process, which justifies an in-depth analysis of these factors [1–3].

Researchers’ interest in trying to measure the influence that certain factors have on the attitudes of students or teachers has given rise to numerous investigations regarding which measurement instruments such as the Thurstone type, Likert scales, or questionnaires are most adequately designed and validated. In the analysis of social behavior such as attitudes toward mathematics, it is necessary to apply a correct sampling methodology and use both a reliable instrument to measure behavior and carefully interpret the results.

The simplicity and the homogeneity of the Likert scale have made it the most frequently used instrument in the measurement of attitudes and beliefs toward mathematics. Due to the impossibility of measuring the factors that constitute these attitudes and beliefs directly, it is common to construct the variables that represent these factors by adding or averaging the valuations obtained from the items used to measure them.

This scale has been widely used since its publication in 1932 by Likert [4] and is supported by numerous handbooks, such as [5], which are important references in the study of attitudes. There are numerous quantitative studies of attitudes using a Likert scale [6–8], and, for purposes of this study, attitudes regarding mathematics [9–12]. However, when constructing the factors to be measured, the linearity of the scale is implicitly imposed, and it is assumed that all the items have the same weight.

When the Likert scale is designed to explain a set of factors that can explain the objective factor of study, like attitude in the scale of Auzmendi [13], there is usually a high association between them, which can make it difficult to design and assign the items to the factors. In these cases, validation is usually carried out by consulting a panel of experts and analysing the reliability by calculating parameters such as Cronbach's alpha coefficient. However, the high association among the factors can generate an overestimation of these parameters and induce an error in the assignment of the items to the dimensional factors of attitude and, therefore, their subsequent estimation.

In this research, a Likert-scale survey that is often used in the study of attitudes is analysed in order to improve the estimation of attitudes students have toward mathematics. Additionally, a new distribution of the items around the attitude factors is proposed based on the conclusions of a panel of experts, with subsequent confirmation by an exploratory factor analysis that confirms that the results found through this methodology cohere with the existing knowledge of the subject.

As mentioned above, it is usual to estimate the factors of an attitude scale by adding or averaging the valuations given to the items that define them [4]. This paper aims to give the keys to the correct design of research using a Likert-scale survey to estimate attitudes toward mathematics and propose a way of estimating the factors under study through a weighted average using the weights obtained in the estimation of the standardized coefficients of the model of structural equations that is used to validate the instrument. This will allow the assignment of greater importance to the items that contain a greater percentage of explained variability for a given factor. As will be seen later, the factors thus constructed are significantly different from those constructed by addition or simple average.

The following section presents the theoretical framework, introducing the summative character of the Likert scale in estimating attitudes toward mathematics as well as the definition of attitude. A brief review of the most frequently used instruments for estimating it is also made, as well as its use in the scientific literature. Next, the objectives of this work are indicated, the methodology followed is shown, and the samples and the instrument to which it has been applied are presented. Finally, the most important results and conclusions are detailed.

## Theoretical framework

The elaboration of instruments for the measurement of attitudes through ordinal items, as proposed by Likert [4], has been widely used in the scientific literature in the social sciences and psychology for the estimation of constructs that are not directly measurable. Many handbooks establish the summative construction of a Likert scale in order to estimate complex variables like attitude [5].

Likert proposed the estimation of the objective factors of study in the scale through the sum of the scores of the different items. This is assumed by researchers who use such scales; proof of this is termed by authors such as Cooper, Blackman and Keller [14] or Krosnick, Judd, and Wittenbrink [15] as "Likert's method of summated ratings".

Since the middle of the past century, the construct known as “attitude” has been widely studied in various lines of research in the fields of psychology and education. However, McLeod [16] brought a new vision to the field and contributed to the concept of attitude being part of the affective factors considered in the study of the affective domain in mathematics education. Traditionally, the objective of these investigations was to develop quantitative methods for measuring attitudes toward mathematics and analysing the relationship among these and other characteristics of the participants such as academic performance, gender, age, or level of education. This was the reason these studies are characterized by not specifying the definition of attitude or, in many cases, by defining it in terms of the instrument that is being used to measure it [2].

Although there is no unanimity with regard to the definition of attitude, there is certain agreement to consider it as consisting of modifiable mental states which, therefore, can be influenced. Hart [17] and Gómez-Chacón [18] agree in understanding attitude as an evaluative predisposition that determines personal intentions and influences behavior. Attitudes are characterized by appearing at any age, although they tend to be positive at younger ages; they can be positive toward one part of the subject and negative toward another, and they are adjustable according to their intensity and are reflected in the predisposition to the subject through feelings toward the teacher or a specific type of activity.

The studies of Auzmendi [13] and Gómez-Chacón [18] defend the position that attitude consists of three components: cognitive, affective, and behavioral. The cognitive component refers to conceptions and beliefs with respect to the people, objects, or contexts in which we live; the affective component refers to the emotions and feelings that these people, objects, or contexts awaken—they can be both pleasant and unpleasant. Finally, the behavioral component is related to the behavior shown in reaction to certain stimuli.

The review of the literature gives us a list of numerous validated instruments for measuring attitudes toward mathematics such as the Thurstone type, Likert scales and other questionnaires. Among the most frequently used Likert scales for the measurement of attitudes toward mathematics we can find:

- The E (Enjoyment of mathematics) and V (Value of mathematics) scales designed and validated by Aiken [19] that respectively measure the enjoyment of mathematical concepts, symbols, and applications using a computer, as well as the importance of the subject. They consist of 12 and 11 items each.
- The Scale of Attitudes toward mathematics designed and validated by Fennema and Sherman [20], formed initially by four subscales that contain 12 items each and that try to measure, among others, the confidence, utility, and perception of the teacher toward the subject. Later studies have updated the expressions and vocabulary of the items and reduced them from 48 to 47.

At the Latin American level, the most widely used scale for measuring the attitudes of secondary school students [13, 21], university students [22–26], and teachers [27] is the scale that is analyzed in this study: the Likert-type scale of attitudes toward mathematics developed by Auzmendi [13].

## Objectives

For the development of a measurement of attitudes regarding mathematics and the validation of the results, it is important, in the first place, to use a panel of experts to create the items. Next, the instrument should be applied to a pilot sample in order to analyse its validity and reliability. In the event that the instrument serves to estimate multiple factors of attitude, an exploratory factor analysis [EFA] should be carried out on the pilot sample to allow a definition of the grouping of the items. This distribution of items must be verified by a confirmatory factor analysis [CFA] on a larger sample [28]. This analysis is even more important if, as in this case, there is a high association among the different factors estimated with the instrument.

The purpose of this study is to obtain a better estimation of the factors of attitude toward mathematics, substituting the usual sum of the item scores by their weighted average with weights depending on the importance the item has for the factor it measures. This methodology was used to analyse the results obtained through Auzmendi’s scale of attitudes toward mathematics [13]. The specific objectives are:

- Obtaining a more adequate reordering of the items that make up the attitudes scale, motivated by the application of an EFA and the analysis made of the statements of the items in the expert panel consultation.
- Measurement of the degree of association among the five factors proposed by Auzmendi for the measurement of attitudes toward mathematics since a high degree would justify the value of consistency obtained by the author as well as its overestimation.
- Obtaining the relevance that each item has in the explanation of its corresponding factor so that the weighted means of factor valuation can be calculated.
- Comparison of the values obtained for the simple average of the factors with the original and new ordination, as well as with the weighted average.

This will allow obtaining more adequate estimates of the dimensions of the attitude toward mathematics using the Auzmendi scale. However, this methodology could also be applied to improving the estimates of other attitude scales and other complex behavioural variables.

## Method

### Participants

The participants in this research were students of different grades from the University of Cordoba (n = 1293) during the 2014/2015, 2015/2016 and 2016/2017 academic years. The sampling was carried out in two phases—in the first, 408 students were surveyed to obtain a pilot sample, and there were 885 students in the second sample; for a total of 1293 students.

The total sample was composed more women (64.70%) than men and the average age was 20.36 (s = 3.34). They were all adult when they completed the questionnaire. The surveys were conducted in the facilities where they usually receive teaching randomly, anonymously, and voluntarily, and the testing did not request personal data. Each answer was coded with an alphanumeric code to identify the degree and number of the questionnaire.

Firstly, and according to the guidelines of the World Health Organization, students were given a document for their consent. This document was read aloud and all doubts and comments raised by the students were resolved. Then they were given the questionnaire and were asked to assess their desire to complete it, for which they had all the time they needed and were assured that non-participation would not have any negative effect.

### Data collection instruments

The instrument that was applied was Auzmendi’s scale of attitudes toward mathematics [13]. This questionnaire was originally validated on a sample of 1221 Spanish students and consists of 25 questions on a Likert scale, with 15 affirmative and 10 negative statements that allow the following scoring options: Strongly disagree = 1, Disagree = 2, Neutral (Neither agree nor disagree) = 3, Agree = 4, and Strongly agree = 5. They were also asked for information regarding their age, gender, and degree.

Auzmendi [13] selected the items that constituted each of the attitude factors:

- enjoyment, relative to how pleasant is working with the subject;
- anxiety, referring to the feeling of fear and discomfort that the student manifests vis-à-vis the subject;
- utility of mathematics, or value that the student considers the acquisition of mathematical knowledge will have for their academic or professional future;
- motivation regarding the study and use of the subject in their studies or in daily life;
- confidence in their own mathematical ability.

The items are grouped into these factors as shown in Table 1.

**Table 1:** Group of items by dimensional factor [13].

Dimensional factor	Items
**Utility**	1, 6, 15, 16, 19 and 21
**Anxiety**	2, 3, 7, 8, 12, 13, 17, 18 and 22
**Enjoyment**	4, 9, 14 and 24
**Motivation**	5, 10 and 25
**Confidence**	11, 20 and 23

A preliminary analysis carried out by eight experts in: development of data collection tools (2), mathematics didactics (3), psychology (1), and pedagogy (2) revealed that some of the items could show statements that were not associated with the factors proposed by Auzmendi. Examples include:

- Item 15, placed by Auzmendi in the Utility factor: "I hope to use little mathematics in my professional life" refers to the hope that the interviewee may have about not using mathematics but not the belief of its utility. Therefore, this item could be included in a more adequate way in the Anxiety factor.
- In a similar way, item 19 can be analysed: "I would like to have a job in which I had to use mathematics," which, once again, was included in the Utility factor, can motivate a response more related to the enjoyment that the interviewed experiences when working with mathematics than the perception of utility towards this subject.

These findings motivate the need to restructure the items so that the information collected is directed to explaining the underlying factors. A strong relationship between the five dimensional factors that explain the attitudes toward mathematics would demonstrate that the consistency of the questionnaire obtained by the author was high even though some items might not be correctly associated with their factor.

### Statistical analysis

Once the data were collected they were processed, the results of the statements were reversed with a negative sense (items 2, 5, 7, 10, 12, 15, 16, 17, 22 and 25), and were analyzed with the SPSS programs [29] for the estimation of the factorial analysis and the calculation of factor values, AMOS [30], for the estimation of the structural equation and R for the calculation of the omega coefficients of consistency.

To analyze the latter, both the Cronbach's alpha coefficient and the Omega coefficient for the instrument were calculated globally with values of 0.896 and 0.901, and subsequently for each factor originally defined by Auzmendi [13] (Table 2). Omega coefficients were calculated because the number of items affects the Cronbach's alpha values [31]. This coefficient is less sensitive to the number of items in each dimension [32]. We can observe that the global internal consistency is very high, close to 0.9 for both coefficients. All the constructs can be considered consistent with the exception of Confidence, whose value is excessively reduced.

**Table 2:** Cronbach’s alpha and omega per factor.

Component	Cronbach’s Alpha	Omega
**Enjoyment**	0.827	0.857
**Anxiety**	0.866	0.881
**Motivation**	0.678	0.723
**Utility**	0.706	0.740
**Confidence**	0.555	0.594

Once the sample has been analyzed, the need arises to structure the statistical techniques that will allow us to obtain the objectives pursued in this research. The methodology followed was distributed in three steps:

Step 1. Due to the possible inconsistencies found in the relationship among some items and their factors, this assignment was improved with a reordering based on a double validation—on the one hand, an EFA on a pilot sample of 408 students and, on the other hand, consultation with a panel of experts.
Step 2. The sample was expanded to 1293 students and a CFA was applied and validated, estimating a model of structural equations as proposed by authors such as Worthington and Whittaker [28]. This technique improves the EFA since relationships among the different factors measured through the instrument can be imposed in their definition.
Step 3. The creation of the SEM allowed us to estimate, on the one hand, the degree of association among the different factors and, on the other, the weight that the response to each item has on the estimation of its corresponding factor in the following way:

The equations of a SEM can be denoted as

η=Bη+Γξ+ζ (1)

where *η* represents the factor in a model, *B* represents the parameter coefficients that link factors with other factors, *Γ* is the corresponding matrix of parameter coefficients linking the exogenous variables (ξ), which are the items, with the factors; and *ζ* represents the error in the prediction of *η*. Therefore, the matrix measures the relevance that each item has for the factor that it estimates and the value is comparable with that of other items due to the homogeneity of the scale. However, standardized coefficients were used to construct the weights.

The latter allowed us to compare the estimate obtained for each factor with the simple average of the scores of the items that explain them and those obtained by weighing the response of each item with its relevance in its explanation [33].

In order to obtain a more adequate classification of the items, a factorial analysis was proposed by the principal component method with Oblimin rotation, taking as a criterion for extraction obtaining a value higher than the unit in the associated eigenvalue.

The model of structural equations was defined from the configuration obtained in the factor analysis including all the possible correlations among factors, which allowed us to determine which are relevant [34].

Finally, after making the two estimations of the factors—the weighted average and the simple average—their normality was analyzed using the Kolmogorov-Smirnov test and, after discarding its compliance, the application of the Wilcoxon range test, which allowed to decide if the differences between the estimated factors of the two forms were significant. The effect size was calculated with Cliff’s delta method for ordinal variables [35].

## Results

### Exploratory factor analysis

To analyze the applicability of the factorial analysis for the pilot sample of 408 students, the asymmetry and pointing coefficients of the variables were calculated resulting in values between -1.135 and 0.385 for the first and between -1.228 and 1.654 for the second variable so that the distributions are not far in excess of the normality hypothesis. However, remember the ordinal character of the variables and, therefore, the impossibility of the variables to follow this distribution; but the asymmetry and pointing values obtained, together with the high sample, allow the applicability of the technique. Due to the nature of the variables, the analysis of the presence of atypical values is not necessary.

The model that was finally considered in the analysis was constructed from 22 items since items 11, 16, and 20 were removed due to their reduced communalities, which affected it negatively. The Kaiser-Meyer-Olkin measure, with a value of 0.901, and the Bartlett sphericity test, with a p-value lower than 0.001, demonstrate the existence of an association among the items and, therefore, the suitability of the applicability of the EFA.

The 22 variables finally considered were grouped in five dimensional factors with a total variance explained to 61.94% by means of the principal component method, to which an Oblimin rotation was applied. Table 3 shows the commonalities associated with these variables, as well as the factorial loads, whose absolute value is greater than 0.3, associated with each of the five factors.

**Table 3:** Exploratory factor analysis results.

Item	Communalities	Factors				
		1	2	3	4	5
**P04**	0.722	0.834				
**P09**	0.666	0.782				
**P14**	0.743	0.695				
**P24**	0.534	0.617				
**P19**	0.553	0.594				
**P17**	0.704		0.796			
**P02**	0.683		0.794			
**P22**	0.726		0.774			
**P07**	0.645		0.761			
**P12**	0.597		0.626			
**P15**	0.555		0.626			
**P05**	0.686			0.858		
**P10**	0.607			0.676		
**P25**	0.581			0.668		
**P13**	0.698				0.747	
**P08**	0.693				0.741	
**P18**	0.581				0.681	
**P03**	0.440				0.510	
**P23**	0.471				0.471	
**P21**	0.612					-0.803
**P06**	0.548					-0.667
**P01**	0.569					-0.649

As can be seen, the items have been ranked in decreasing order based on their factorial loads with respect to their corresponding factors. These exceed the value of 0.6 except the last of factors 1 and 4. All commonalities are also higher than 0.4 and most of them exceed 0.6.

In this way, the new assignment of items to factors carried out in the EFA is as shown in Table 4, which also specifies the consistency values given by the Cronbach's omega and alpha coefficients as well as the information previously presented for the Auzmendi scale for comparison.

**Table 4:** New proposal of grouping in dimensional factors.

Factor	Auzmendi distribution			Proposed distribution		
	Items	Alpha	Omega	Items	Alpha	Omega
**Utility**	1, 6, 15, 16, 19 y 21	0.706	0.740	1, 6 y 21	0.650	0.685
**Anxiety**	2, 3, 7, 8, 12, 13, 17, 18 y 22	0.866	0.881	2, 7, 12, 15, 17 y 22	0.868	0.891
**Enjoyment**	4, 9, 14 y 24	0.827	0.857	4, 9, 14, 19 y 24	0.841	0.868
**Motivation**	5, 10 y 25	0.678	0.723	5, 10 y 25	0.678	0.723
**Confidence**	11, 20 y 23	0.555	0.594	3, 8, 13, 18 y 23	0.802	0.833
**TOTAL**		0.896	0.901		0.909	0.910

Six of the 25 items have been redistributed by the EFA so that four of them go from explaining the anxiety that the interviewees show toward mathematics to explain their confidence. In addition, items 15 and 19 were taken from the group that explains the utility of this subject and included in Anxiety and Enjoyment, respectively. Finally, items 11, 16, and 21 have been excluded from the analysis due to their low explanatory capacity.

When the items are reorganized in this way, the global consistency remains practically invariant. Only an improvement of one tenth is observed, and the values of the omega and alpha coefficients of Cronbach improve for all the factors except for Motivation, which remains without changes since this factor stays invariant in this transformation; and the Utility factor, in which it is slightly reduced—from 0.706 to 0.650 in the case of the alpha coefficient, and from 0.740 to 0.685 in the case of the omega coefficient. It is worth highlighting the increase in the consistency of the Confidence factor, which, as happened in Auzmendi [13], showed an excessively low value of alpha, equal to 0.555, which has risen to 0.802 (from 0.594 to 0.833 in the case of the omega coefficient).

The results support the hypothesis regarding the necessary redistribution of the items of the questionnaire both from the point of view of the interpretation of the statements by the panel of experts and due to the improvement obtained for the internal consistency of the instrument for this sample.

Next, we show the estimation of the structural equation created for the total sample formed by 1296 students, which confirms the factorial analysis estimated in the previous step, and from which the weights of each item will be estimated in the calculation of the weighted averages for the estimation of the factors.

As previously mentioned, the estimation of the standardized coefficients of the regression between the items and the factors to which they are associated according to the previous classification allow us to estimate these factors as the weighted average of the values given to each of the items in its category.

### Structural equation

**Fig 1:**
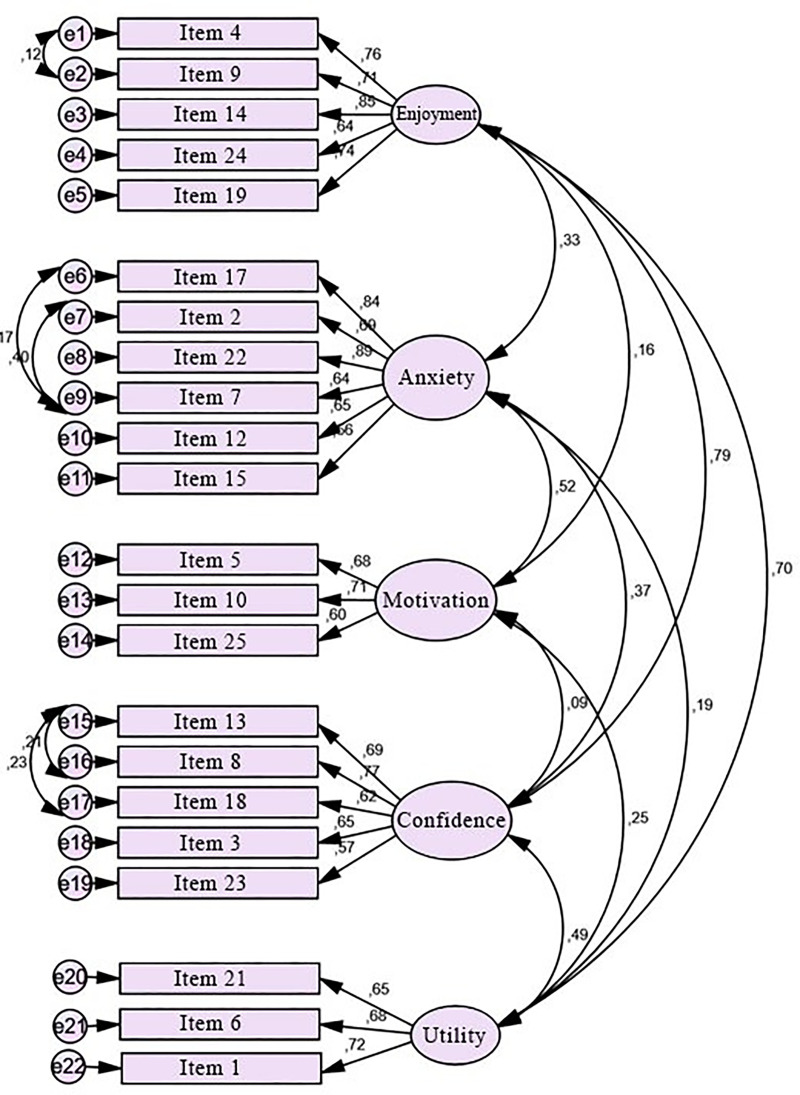
Estimated structural equation explaining the factors with their proposed distribution.

Fig 1 shows the structural equation estimated by the maximum likelihood method for the 22 items considered, the five-dimensional factors, and the organization found in the previous section. The names of the items identify each factor in Auzmendi’s original scale.

The absolute, incremental, and parsimony adjustment measures obtained for this model, as well as the widely accepted criterion of goodness, are shown in Table 5. All of them, together with the relevance of all the variables considered and the value of the residuals, show the validity of our model.

**Table 5:** Adjustment measures.

Adjustment measures (absolute)		Value	Optimal value
Goodness of fit index	GFI	0.957	> 0.9
Root Mean Square Error of Approximation	RMSEA	0.042	< 0.05
P-value of close fit	PCLOSE	1.000	> 0.05
Standardized root mean square residual	SRMR	0.039	< 0.08
**Incremental adjustment measures**			
Normed fit index	NFI	0.948	> 0.90
Comparative adjustment index	CFI	0.963	> 0.90
**Parsimony adjustment measures**			
Chi-squared test for normality	NCS	3.312	Values between 1 and 3

Once the structural equation was estimated and validated, we analyzed the correlations among the five factors: Enjoyment, Confidence, Utility, Anxiety, and Motivation. The results are shown in Table 6. All the relationships are significant; the strongest relationship is observed between the Enjoyment and Confidence factors, which indicates that a greater enjoyment for mathematics facilitates greater confidence in the subject and vice versa. At the other extreme are Motivation and Confidence, which, while significant, have a reduced value that indicates that, although motivation by itself could generate confidence, other factors can influence it.

**Table 6:** Correlations between dimensional factors.

Relation		Estimated values		Relation		Estimated values	
Enjoyment	<—>	Anxiety	0.330	Anxiety	<—>	Confidence	0.368
Enjoyment	<—>	Motivation	0.158	Anxiety	<—>	Utility	0.189
Enjoyment	<—>	Confidence	0.786	Motivation	<—>	Confidence	0.095
Enjoyment	<—>	Utility	0.698	Motivation	<—>	Utility	0.253
Anxiety	<—>	Motivation	0.520	Confidence	<—>	Utility	0.488

Also noteworthy, due to their intensity, are the relationships that indicate that a high perception of mathematics utility increases the enjoyment level shown for this subject. In the same way, a lower level of anxiety is associated with a greater feeling of confidence. It is important to remember that the responses to the items, presented in a negative way, were inverted at the beginning of the study to represent positive values for the attitudes toward mathematics.

### Estimation of factors

Once the model of structural equations was built, the five factors, with their proposed distribution, were estimated in two ways—in the first one, through the simple arithmetic mean of the values obtained in each item. In the second, from the estimations of the standardized coefficients of the regression, which are shown in Fig 1, we calculated the means weighted with these as weights. The results of these coefficients are shown in Table 7. The differences between the standardized coefficients in some factors are high, as in items 15 and 22 of the Anxiety factor, or 8 and 23 of Confidence.

**Table 7:** Standardized regression coefficients.

Item	Estimation	Factor	Item	Estimation	Factor
P04	0.764	Enjoyment	P05	0.682	Motivation
P09	0.706		P10	0.714	
P14	0.853		P25	0.600	
P24	0.637		P08	0.767	Confidence
P19	0.744		P18	0.619	
P17	0.845	Anxiety	P03	0.655	
P02	0.695		P23	0.574	
P22	0.886		P13	0.694	
P07	0.641		P06	0.683	Utility
P12	0.649		P01	0.724	
P15	0.560		P21	0.646	

The high variability of the coefficients shows the need to assign different weights to the items of the same factor. Once the five factors were estimated, a transformation was made to present them on a scale between 0 and 100 for a better interpretation of the results. A descriptive study is shown in Table 8, in which there are high differences between the calculated values with the scale originally given by Auzmendi and the one proposed in this research, except for the Motivation factor, which, as seen above, contains the same items in both distributions, and in which the weights do not show strong discrepancies among the items.

**Table 8:** Statistical descriptions of the different ordinations.

Factor	Original distribution Simple average		Proposed distribution and Simple mean		Proposed distribution and weighted mean	
	Mean	Standard deviation	Mean	Standard deviation	Mean	Standard deviation
**Enjoyment**	38.7950	22.3796	39.2537	22.0119	39.5607	22.1178
**Anxiety**	50.9070	19.2630	51.3631	23.4481	51.3515	23.7481
**Motivation**	58.1132	23.3320	58.1013	23.3077	58.2485	23.5198
**Confidence**	71.1404	17.2243	54.8879	20.4933	54.4266	20.7072
**Utility**	56.3684	16.5903	63.3217	20.2998	63.6929	20.2701

When comparing the values of the original distribution factors and the simple means with those of the proposed distribution and weighted averages, it was observed that the mean values of the factors Enjoyment, Anxiety, and Utility were being underestimated with the previous methodology, while the average value of the Confidence factor was clearly overestimated.

Finally, we analysed, through the Wilcoxon distributions comparison contrast, the existence of significant differences among the factors. The results are shown in Table 9. We opted for a non-parametric contrast, due to the absence of normality of some of the constructed variables, analysed with the Kolmogorov-Smirnov test. The effect size of differences was computed through the Cliff’s delta statistic. A value smaller than 0.11 is considered very small; values between 0.11 and 0.28 are considered small; values between 0.28 and 0.43 are considered medium; and values greater than 0.43 are considered large [35].

**Table 9:** Results of p-values of Wilcoxon contrasts and Cliff’s delta effect size for ordinal variables.

Factor	Original distribution vs. Proposed distribution and weighted mean		Original distribution vs. proposed distribution and simple mean		Proposed distribution and simple mean vs. proposed distribution and weighted mean	
	p-value	Effect size	p-value	Effect size	p-value	Effect size
Enjoyment	0.019	0.021	0.019	0.013	<0.001	0.025
Anxiety	0.029	0.007	0.055	0.006	0.806	0.000
Motivation	<0.001	0.017	-	-	<0.001	0.017
Confidence	<0.001	0.475	<0.001	0.469	<0.001	0.026
Utility	<0.001	0.286	<0.001	0.247	<0.001	0.075

Looking at the size of the effect, the differences are large for the Confidence factor and medium for the Utility factor.

All the factors estimated with the weighted mean and the proposed distribution of the instrument are significantly different from those obtained with the simple mean and the distribution of Auzmendi except Motivation. Significant differences have also been found between the two simple averages of the factors Confidence and Utility and, to a lesser extent, Enjoyment and Anxiety. Finally, the differences between the two means calculated with the proposed distribution are also significant when measuring Enjoyment, Motivation, Confidence, and Utility. In this last case, the calculation of the simple mean underestimates the values of Enjoyment, Motivation, and Utility, while Confidence was overestimated.

## Conclusions

When carrying out any attitudes study with the use of an information collection instrument, both the correct calibration of the measurement instrument and the calculation of the factors to be estimated from the items are of vital importance. The incorrect use of any of them can lead to erroneous conclusions.

This paper reviewed the methodology for the treatment of a widely used measurement instrument for attitudes toward mathematics, with which the items have been analysed using the five factors that define the attitudes. At first, a new ordering of items was proposed through the analysis of internal consistency—the elaboration of an EFA on a pilot sample, which justified the initially proposed changes. Then, a CFA confirmed the consistency of the results obtained. Especially important is this last step in the attitude measurement, a multi-component instrument, due to the possible relationship among them, usually not addressed in the EFA. The existence of this relationship has been confirmed in the developed case as well.

The need to question the suitability of constructing attitude factors toward mathematics as the sum or simple average of the responses to the items, a method proposed by Likert [4] and widely used in the literature, has been raised not only for attitudes toward mathematics but for those in which Likert-type scales are used to estimate other factors. The existence of significant differences in the estimation of the factors has been demonstrated through the simple average of the item scores and a weighted average based on the relevance of the items in the estimation of each factor.

The estimation of non-measurable factors such as confidence or utility through the sum of the values of each item or the simple average imposes the condition that all the responses to the items have the same relevance in the explanation of the factor regardless of the content of the question. The coefficients of the structural equation between the factors and the items or the commonalities of the factorial analysis can be measures of the importance that each item has.

This proposed methodology for improving the estimation of attitudes toward mathematics was applied to the survey developed by Auzmendi, finding significant differences in the estimation of a part of the estimated factors, which are large in the Confidence and Utility factors. In the first, an overestimation is generated and in the second, an underestimation.

The aforementioned has been able to bring about conclusion that, in the investigations carried out in recent years on attitudes toward mathematics, higher evaluations have been obtained with respect to the feeling of confidence regarding one’s ability in mathematics than those that the participants in these studies actually possess. Examples in which it could have occurred are in the studies by Auzmendi [13] (83.93 points out of 100), Flores and Auzmendi [25] (72.20 points out of 100), or Nortes Martínez and Nortes Checa [26] (78.80 points out of 100).

In the same way, the interviewees could perceive that mathematics is more useful for their professional and academic life than the attitude that was obtained in these previous investigations (67.93 [13], 66.13 [25], 67.80 [26] points out of 100 respectively).

For example, in the works mentioned above, a poor estimate of these factors could have led these studies to draw erroneous conclusions about the correlations among levels of attitude and variables such as gender, course, or high school modality, as well as the differences found between men and women or among students who study different university subjects. Although in the rest of the factors the effect size is small, the differences—significant—cause important variations in the estimation of the mean and the standard deviation of the factors.

Descriptive studies that explore the attitudes toward mathematics of students of different gender, ethnicity, geographical situation, or who are studying different subjects, aim to provide information to mathematics teachers so that they can carry out interventions that help their students improve. Some works have already been developed along this line such as Hannula et al. [2], but it is necessary to continue working on this because very little progress has been made and poor results have been obtained. It is especially important that each of the attitude factors are estimated correctly to avoid reaching incorrect conclusions.
